# Asymptomatic Glandular Odontogenic Cyst of the Right Posterior Maxilla Involving the Maxillary Sinus: Case Report With Literature Review

**DOI:** 10.7759/cureus.87924

**Published:** 2025-07-14

**Authors:** Mohammed H Albodbaij, Bander Y Alkarri, Israa A Alameer

**Affiliations:** 1 Oral and Maxillofacial Surgery, King Fahad Hospital, Ministry of Health, Hofuf, SAU; 2 Dentistry, Primary Health Care Corporation, Hofuf, SAU

**Keywords:** caldwell-luc approach, glandular odontogenic cyst, impacted teeth, maxilla, maxillary sinus

## Abstract

Glandular odontogenic cyst (GOC) is a rare jaw cyst with potential for recurrence and aggressive behaviour. We present a case of a 41-year-old male patient with an incidentally discovered, asymptomatic unilocular radiolucency in the right posterior maxilla extending into the maxillary sinus, along with a focused literature review on GOC cases involving the maxillary sinus. Initial radiographic diagnosis suggested an odontogenic keratocyst (OKC). The lesion was surgically enucleated via Caldwell-Luc approach, and histopathology confirmed GOC characterized by mucous cells, epithelial plaques, and pseudostratified columnar epithelium resembling respiratory type. The postoperative course was uneventful, with satisfactory osseous healing at six months. This case underscores the need to consider GOC in differential diagnoses of sinus-involved maxillary lesions. Imaging and histology are critical for diagnosis, and long-term follow-up is advised.

## Introduction

Glandular odontogenic cyst (GOC) is a rare developmental cyst first described by Padayachee and van Wyk in 1987 as a 'sialo-odontogenic cyst', owing to the presence of mucous cells and mucin pools in its lining [1]. Gardner et al. later proposed the term 'glandular odontogenic cyst', emphasizing its odontogenic origin in the absence of salivary gland tissue [2]. It was officially recognized by the World Health Organization (WHO) in 1992 and included in the latest WHO classification of odontogenic lesions in 2022 [3].

GOC predominantly affects the anterior mandible and is approximately four times more common in the mandible than in the maxilla. It typically presents in middle-aged individuals, with a slight male predilection [4-7]. Most cases are asymptomatic and exhibit slow growth [6]. Radiographically, GOC presents as a well-defined unilocular or multilocular radiolucency, often exhibiting scalloped borders [6]. In larger lesions-particularly in the maxilla, where cortical bone is thinner-adjacent anatomical structures may be involved.

The diagnosis of GOC remains challenging due to its variable histological features and radiographic similarity to other odontogenic cysts such as odontogenic keratocyst (OKC), lateral periodontal cyst (LPC), and even low-grade central mucoepidermoid carcinoma (CMEC) [7-9]. Therefore, accurate diagnosis requires a combination of imaging, histopathology, and clinical correlation.

Kaplan et al. were the first to propose a set of major and minor histopathological criteria to aid in distinguishing GOC from similar lesions [10]. These criteria were later refined and validated by Fowler et al., who conducted one of the largest multicenter reviews including 46 confirmed cases [11]. Their analysis emphasized key features such as mucous cells, epithelial spheres, and microcyst formation as essential for diagnosis.

This report presents an unusual case of an asymptomatic GOC in the right posterior maxilla extending into the maxillary sinus, discovered incidentally on routine radiography. Given the lesion's rarity and diagnostic complexity, we also provide a comprehensive literature review to highlight comparable cases, diagnostic challenges, and treatment strategies.

## Case presentation

**Figure 1:**
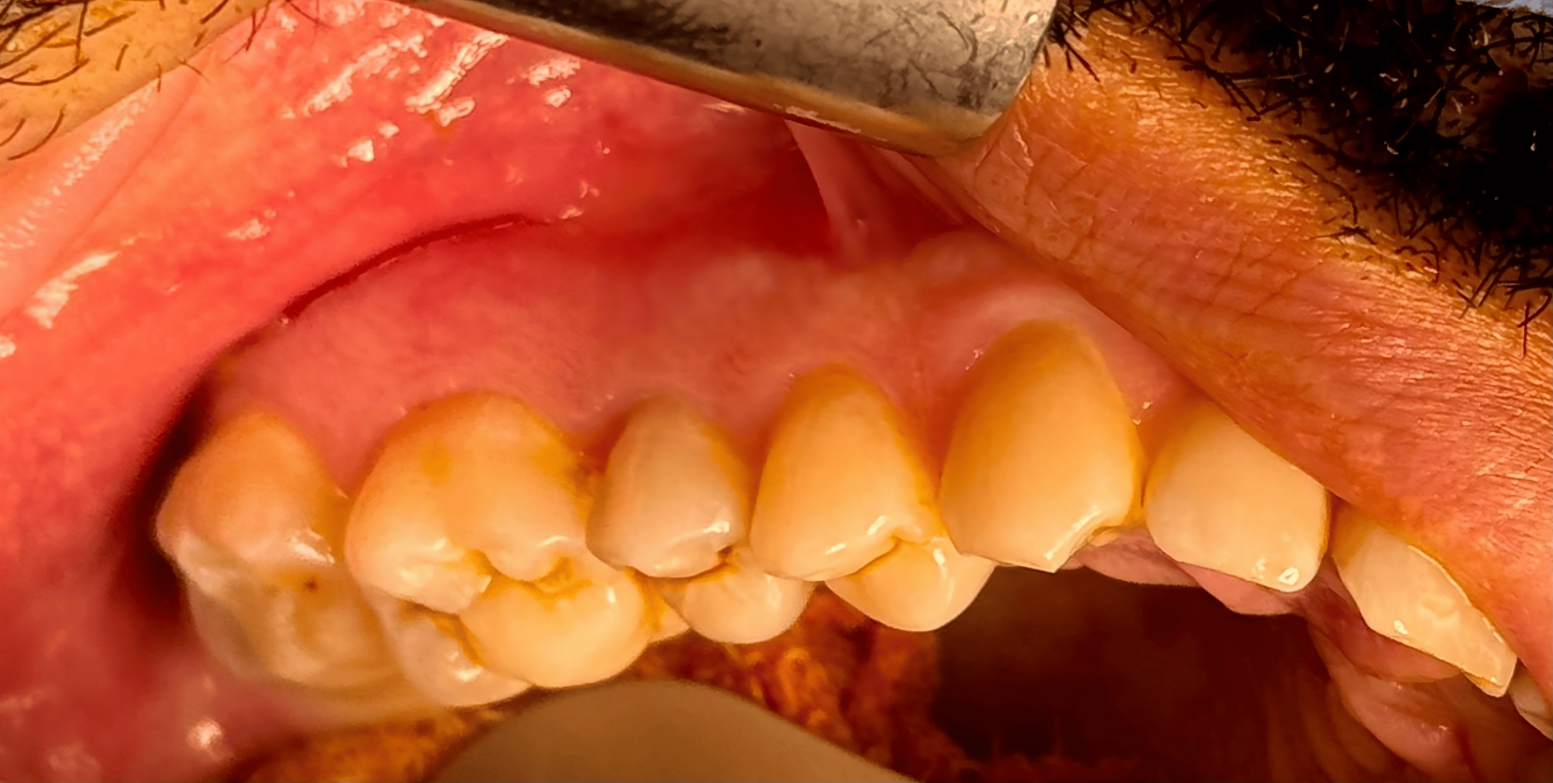
Intraoral clinical photograph showing the right posterior maxilla with no evidence of swelling or signs of infection

A 41-year-old man was referred to our oral and maxillofacial surgery clinic for evaluation of a radiolucent lesion incidentally discovered in the right posterior maxilla during routine radiographic examination. The patient had no significant medical history except for an allergy to colxicam. He reported a history of surgical extraction of impacted tooth #28, as well as root canal treatment and restorations carried out two years prior. The patient was asymptomatic, with no signs of facial asymmetry, extraoral swelling, or regional lymphadenopathy. Intraoral examination revealed no swelling. Teeth #15, #16, and #17 exhibited no pain or tenderness on percussion. Electric pulp testing indicated normal pulpal vitality (Figure 1).

**Figure 2:**
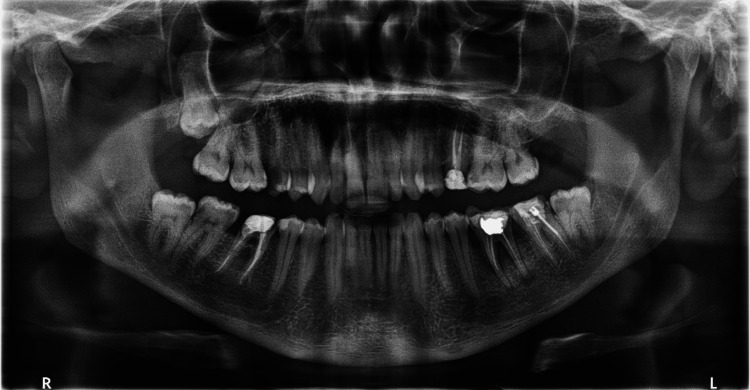
Orthopantomogram showing well defined radiolucency with impacted #18

The panoramic radiograph revealed a unilocular radiolucency with a well-defined border in the right periapical; region, extending mesiodistally from tooth #16 to #18, reaching the maxillary tuberosity. Tooth #18 appeared impacted and displaced, though it was not clearly involved in the lesion. The radiolucency appeared to extend into the right maxillary sinus (Figure 2). Accordingly, a non-contrast CT was advised to eliminate the effect of structural superimposition.

**Figure 3:**
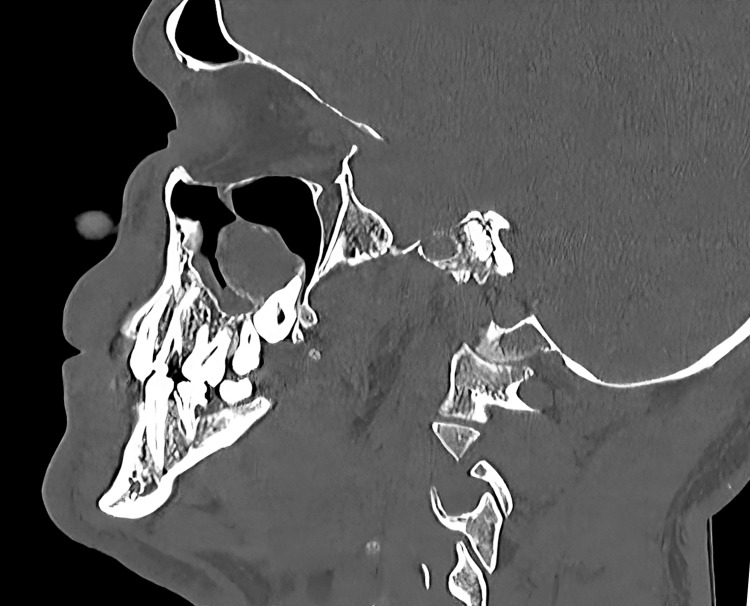
CT sagittal section showing posterolateral perforation and hypodense lesion extended into the maxillary sinus

The CT demonstrated a hypodense lesion with a well-delineated boundary, measuring 3.0 × 3.5 × 2.5 cm, in the right maxilla, associating tooth #18 and extended mesial toward the periapical region of tooth #16 and #17. The lesion extended into the right maxillary sinus, perforating the posterolateral wall. Mucosal thickening was noted at the base of the sinus adjacent to the lesion (Figure 3). No visible connection to the ethmoidal, frontal, or sphenoidal sinuses, or to the nasal cavity, was observed.

**Figure 4:**
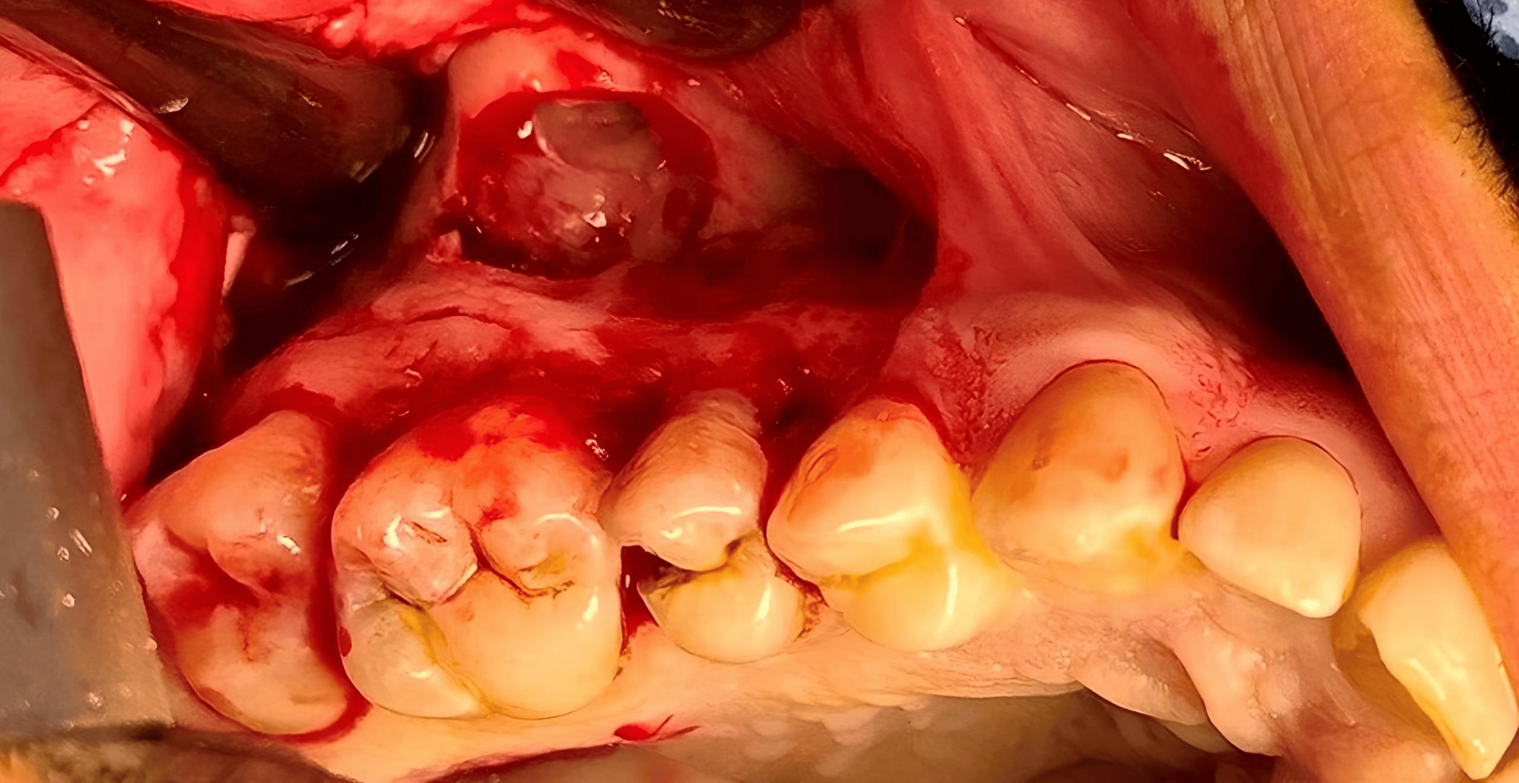
Intraoperative view of the right posterior maxilla showing exposure of the cystic lesion

**Figure 5:**
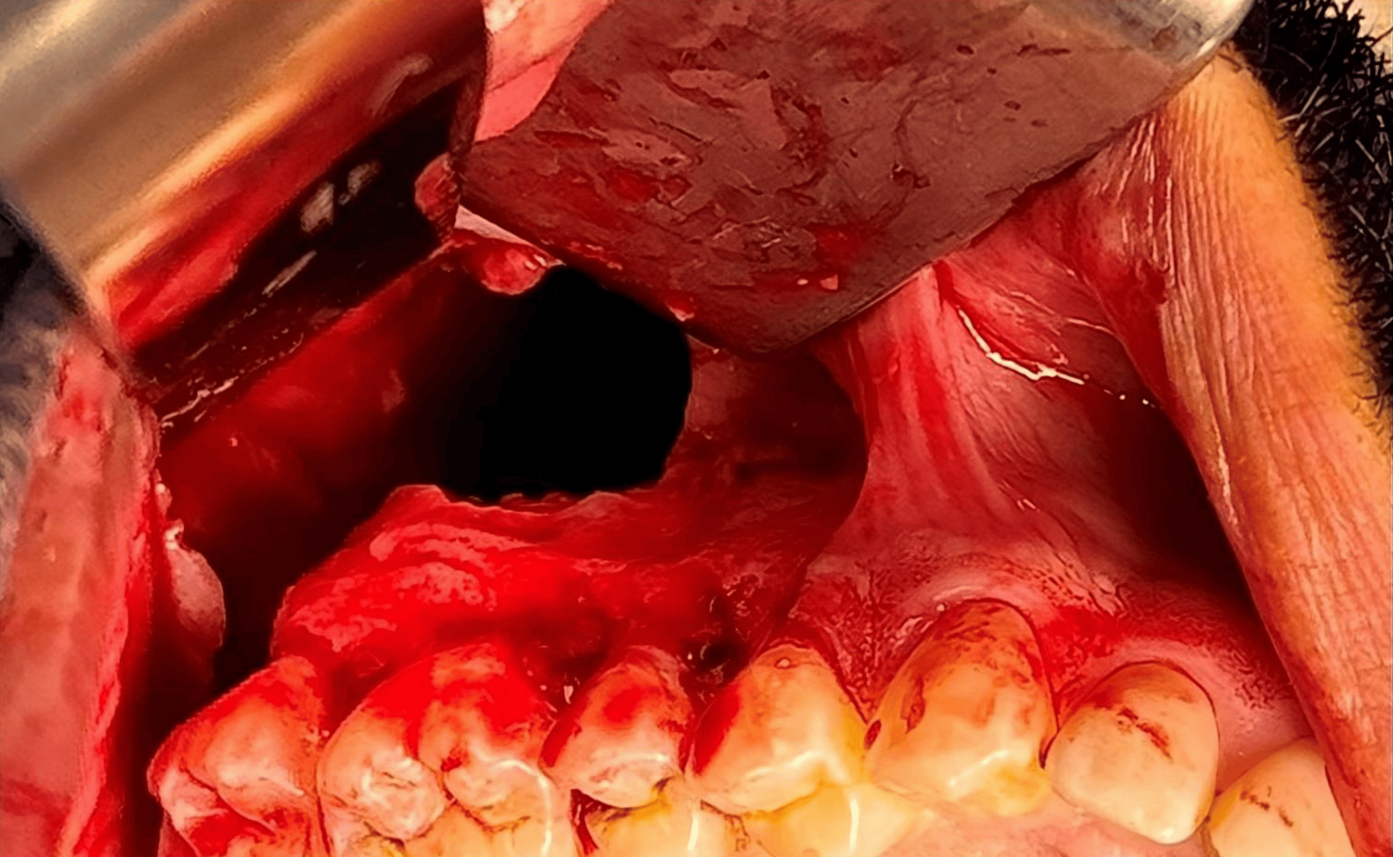
Post enucleation and curettage of the cystic lesion along with the impacted displaced third molar from the maxillary sinus

Enucleation of the lesion was performed using the Caldwell-Luc approach, which included a formal antrostomy through the anterior wall of the maxillary sinus to provide direct access to the lesion. This approach was appropriate due to the lesion’s extension from the periapical areas of teeth #16, #17, and #18, with involvement of the maxillary sinus. Under the administration of general anesthesia with nasal intubation through the right nostril, local anesthesia with 2% lidocaine and epinephrine were infiltrated into the right maxilla. The maxillary sinus was accessed intraorally, distal to the canine region. A mucoperiosteal flap was elevated through a triangular incision made in the gingivobuccal sulcus, just above the tooth sockets from the first premolar to the second molar. To facilitate closure, utmost care was taken to preserve the mucosa adequately. Exposure was extended superiorly, and the anterior wall of the sinus was opened using a round bur to perform an ovoid osteotomy above the tooth roots (Figure 4), the soft tissue lesion was identified, and decompression was performed by aspirating the cystic fluid with a 20-cc syringe. Curettage of cystic legion and surrounding thickened antral lining was performed to ensure complete debridement of sinus cavity. No antral pack was placed, and the sinus was left to heal by secondary intention. The procedure also included removal of the impacted tooth #18 and a partial ostectomy (Figure 5).

**Figure 6:**
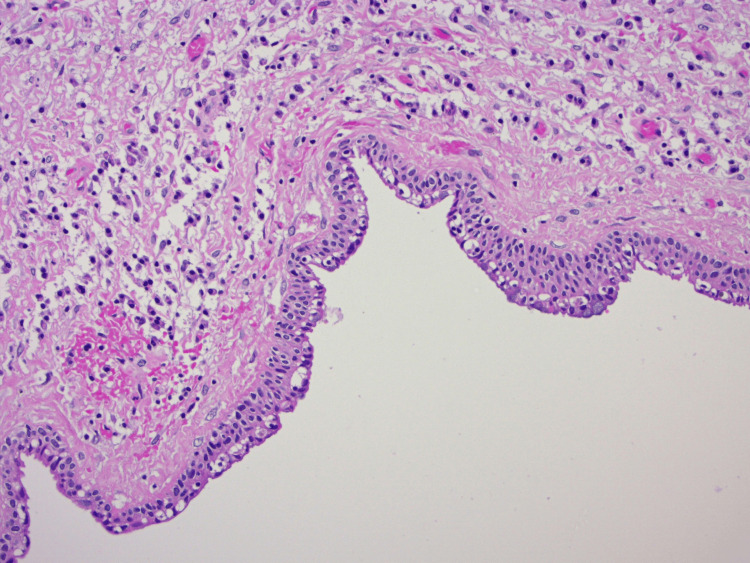
Histopathological image showing cystic lining with a non-keratinized stratified squamous epithelium of variable thickness, focal plaque-like thickening, and scattered mucous cells and mild chronic inflammatory infiltrate in the underlying stroma

A collapsed soft tissue cystic specimen was excised and submitted for histopathological examination. Hemostasis was achieved using suction, and the surgical wound was sutured. Histopathological analysis (Figure 6) revealed a non-keratinized squamous epithelium of variable thickness with epithelial tufting, focal clear duct-like structures, focal epithelial plaques, and mucous cells. The connective tissue wall exhibited collagen fibers, mild inflammatory cell infiltration, and bony trabeculae. Additionally, areas of pseudostratified columnar epithelium resembling respiratory epithelium were observed, consistent with a diagnosis of GOC.

**Figure 7:**
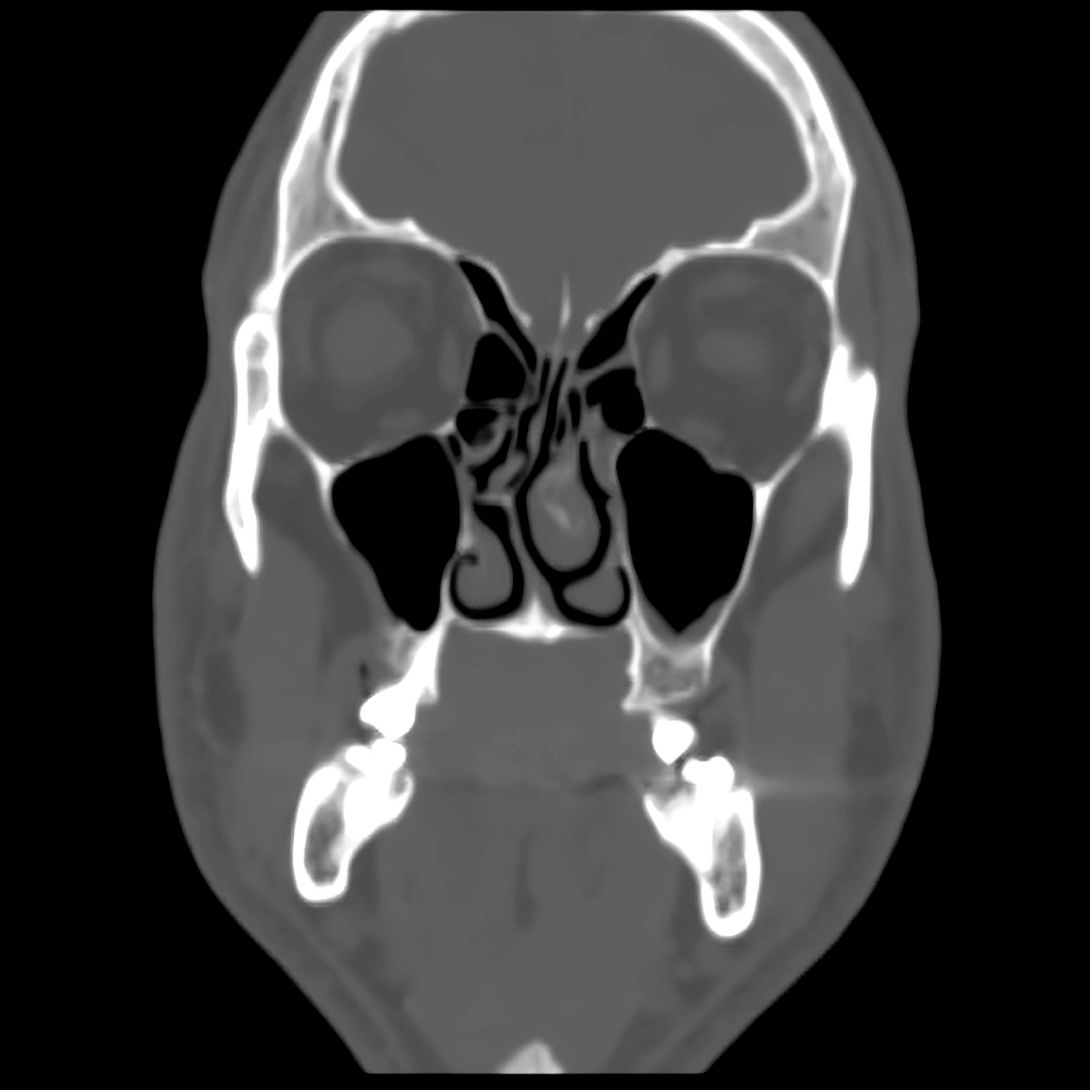
Postoperative CT coronal at six-month follow up showing satisfactory osseous healing

The patient tolerated the procedure well, with no immediate or delayed complications. Postoperative medications included amoxicillin 500 mg and metronidazole 500 mg orally every eight hours for five days, paracetamol 1 g as needed for pain control (maximum 4 g/day), and xylometazoline nasal drops every six hours for 14 days. At the six-month follow-up, the patient was asymptomatic, and CT scan displayed satisfactory osseous healing (Figure 7).

## Discussion

Odontogenic cysts and tumors constitute a diverse group of lesions arising from tooth-forming tissues or their remnants [12]. Cysts involving the maxillary sinus can be intrinsic, such as mucous retention cysts, or extrinsic which are predominantly odontogenic in origin. Despite variation in histological origin, maxillary cysts frequently present with overlapping clinical and radiographic features. Differentiation depends on location, extent of expansion, growth direction, and occurrence of complications. These lesions are often incidentally discovered on radiographic imaging and typically appear as rounded soft tissue masses on the sinus floor [4,12-14].

Water’s view, panoramic radiography, and plain skull radiographs are simple and cost-effective tools suitable for daily clinical use. However, conventional radiographs may not define the extent of maxillary lesions owing to overlapping anatomical structures. Therefore, CT is indispensable, as it allows precise evaluation of lesion size, cortical involvement, and sinus extension [13,14]. GOCs are rare, with a reported frequency ranging from 0.012% to 1.3% of all jaw cysts [12]. This case is rare due to its asymptomatic presentation and unusual location in the right posterior maxilla, extending from the first molar region to the impacted third molar with evident invasion of the maxillary sinus.

A literature review revealed few reports of GOC in the posterior maxilla with sinus extension [4,15,16]. To contextualize the present case, a comparison with previously reported cases of GOC involving the posterior maxilla and maxillary sinus is presented in Table 1.

**Table 1:** Comparative summary of reported cases of GOC in the posterior maxilla with sinus involvement GOC: Glandular odontogenic cyst

Study	Age/Gender		Location					Clinical Presentation			Treatment	Follow-Up Outcome
													Guruprasad et al. [15]	17 / Female		Right posterior maxilla with sinus extension					Asymptomatic swelling			Enucleation, impacted wisdom extraction	No recurrence (period not specified)
Rao et al. [4]	60 / Female		Left posterior maxilla with sinus extension					Asymptomatic swelling			Enucleation	Resolution of symptoms													
													Li et al. [16]	23 / Female		Right Posterior maxilla with sinus extension					symptomatic swelling			Enucleation with marginal ressection	No recurrence at one year
Present study	41 / Male		Right posterior maxilla with sinus extension					Asymptomatic, incidentally discovered			Enucleation, Peripheral ostectmoy	Satisfactory healing at six months													

A multicenter study by Nel et al. analyzing 92 GOC cases found 84% occurred in the mandible, predominantly in the anterior region [17]. Only 6% were in the maxilla, and most of those were anteriorly located, making posterior maxillary involvement exceedingly uncommon [17]. This rarity often leads to initial misdiagnosis as radicular or residual cysts.

Pinheiro et al. presented a rare case of GOC in the posterior maxilla that was initially diagnosed clinically and radiographically as a radicular cyst [18]. The definitive diagnosis was established only after histopathological examination, emphasizing the importance to include GOC in the differential diagnosis of cystic lesions in unusual maxillary sites [18]. Guruprasad et al. reported a GOC case in the right posterior maxilla of a 17-year-old female patient with sinus extension and association with an unerupted third molar [15]. Similarly, Li et al. documented posterior maxillary lesions with sinus invasion requiring more invasive surgical treatment [16]. Manor et al., in an extensive literature review (181 cases), noted that 28% of GOC cases involved the maxilla, with less than 10% localized to the posterior region [9].

Due to the thin bony structure of the maxilla and its proximity to the sinus, lesions in this region can easily expand into the maxillary sinus and attain considerable size without producing clinical symptoms, as also observed in our case [19]. Radiographic features are important for the differential diagnosis of GOC include locularity, radiodensity, and border characteristics [6]. GOC typically appears as a well-defined unilocular or multilocular radiolucency, sometimes with scalloped margins. In aggressive forms, cortical perforation or root displacement may occur [6]. These radiographic features overlap with other odontogenic entities such as radicular cysts, OKCs, ameloblastomas, adenomatoid odontogenic tumors (AOTs), and LPCs [6,8,9,20]. Therefore, definitive diagnosis requires histopathological confirmation. Given the sinus extension in our case, mucous retention cysts were also considered, as their radiographic appearance may mimic GOC [21].

In our case, the lesion was periapical to the molars and involved an impacted third molar. The orthopantomogram failed to delineate pericoronal involvement and antral extension owing to overlapping anatomical structures. The CT scan was crucial in confirming that the sinus extension originated from the odontogenic lesion, rather than a separate pathology. The scan revealed thinning and perforation of the antral floor and posterolateral wall, along with a well-demarcated hypodense ovoid lesion. A vitality test, absence of caries, and no trauma history ruled out radicular cyst or odontogenic sinusitis. Clear sinus extension ruled out mucous retention cyst. Radiographic differentials considered were OKC, ameloblastoma, AOT, LPC, and dentigerous cyst.

Dentigerous cysts typically arise from the follicle of unerupted teeth and commonly occur in the posterior mandible or maxilla involving third molars. AOT predominantly occurs in young females in the anterior maxilla, rarely involving impacted third molars or the sinus, and usually appears as a mixed radiolucent-radiopaque lesion [17]. Odontogenic keratocysts, although more common in the mandibular molar region, may occur in the maxilla with sinus involvement and present as unilocular lesions with scalloped borders [4]. Ameloblastomas, usually seen in individuals aged 20-60 years, may involve the maxillary molars and are often multilocular, although unicystic variants exist [7]. LPCs usually arise in the interradicular canine-premolar region and present as well-defined unilocular or pear-shaped radiolucencies [9].

Histopathological diagnosis is critical for treatment planning, prognosis, and long-term follow-up of GOC [22]. GOC exhibits histological overlap with LPC, particularly its botryoid variant (BOC), dentigerous cysts with mucous metaplasia, and CMEC [6-9]. LPC is lined by thin non-keratinized epithelium with focal epithelial thickenings and glycogen-rich epithelial cells, similar to GOC [6,8]. BOC, an aggressive polycystic variant of LPC, also displays epithelial plaques and clear glycogen-rich cells in a multilobular pattern. Dentigerous cysts usually have thin squamous epithelium, but mucous metaplasia may require consideration [7,8].

Differentiating low-grade CMEC from GOC, especially in its multicystic variant, is critical yet challenging [4,5,8,9,15]. Hence, significant histological overlap exists among GOC, LPC, and CMEC. Fowler et al. and Kaplan et al. proposed widely accepted diagnostic criteria for GOC [10,11]. The WHO classification (2022) considers all histological features as characteristic but not essential. Microscopic criteria include glandular or pseudoglandular structures, cuboidal or columnar cells, and mucin-containing intraepithelial crypts-all of which were evident in our case. Multicystic GOC shows more neoplastic behavior, such as tissue infiltration or daughter cyst formation, compared to unicystic types [10,11].

Treatment options include curettage, enucleation, and resection [9,11,23,24]. While small unilocular lesions respond well to enucleation, larger or multilocular lesions often require more aggressive interventions like peripheral ostectomy or marginal resection [23,24]. Marsupialization followed by secondary surgery has also been suggested for lesions near vital structures [10]. Recurrence rates correlate with lesion size, ranging from 14.4% for small lesions to 85.6% for larger ones, underscoring the need for long-term follow-up [24]. A surveillance period of three-five years is generally recommended owing to the lesion’s aggressive nature and recurrence risk [11,24].

Despite the addition of new knowledge, the case study has a few limitations. The findings are based on a single case, limiting generalizability to a broader population. Advanced imaging methods like MRI or PET scans may provide information on soft tissue involvement or lesion aggressiveness, compared to CT scans. Despite histopathological confirmation, there is an overlap in histology with other cyst types which could impair clinical judgement. The case describes enucleation using the Caldwell-Luc approach but lacks discussion of alternative surgical approaches. 

## Conclusions

Asymptomatic odontogenic cysts or tumors involving the maxillary sinus pose unique diagnostic and therapeutic challenges due to their proximity to vital structures and frequent radiographic overlap with other lesions. This case of GOC is particularly rare due to its posterior maxillary location and silent progression into the sinus. The case underscores the importance of a multidisciplinary approach, combining radiographic, clinical, and histopathological analyses to reach a definitive diagnosis. The lesion’s aggressive potential and recurrence risk necessitate not only precise surgical management but also long-term clinical and radiological follow-up. This report further highlights the critical role of CT in evaluating sinus involvement and guiding the surgical approach. While enucleation via Caldwell-Luc approach was successful in this case, the recurrence potential justifies surveillance of at least three-five years. Clinicians should maintain a high index of suspicion for GOC in atypical presentations, especially in posterior maxillary radiolucencies extending to the sinus.
